# Synthesis of Cyano-Substituted Conjugated Polymers for Photovoltaic Applications

**DOI:** 10.3390/polym11050746

**Published:** 2019-04-26

**Authors:** Mun Ho Yang, Ho Cheol Jin, Joo Hyun Kim, Dong Wook Chang

**Affiliations:** 1 Department of Industrial Chemistry, Pukyong National University, Pusan 48513, Korea; didanagh12@gmail.com; 2 Department of Polymer Engineering, Pukyong National University, Pusan 48513, Korea; rideocnd123@gmail.com

**Keywords:** conjugated polymers, cyano group, polymer solar cells

## Abstract

Three conjugated polymers, in which the electron-donating (D) 5-alkylthiophene-2-yl-substitued benzodithiophene was linked to three different electron-accepting (A) moieties, i.e., benzothiadiazole (BT), diphenylquinoxaline (DPQ), and dibenzophenazine (DBP) derivative via thiophene bridge, were synthesized using the Stille coupling reaction. In particular, the strong electron-withdrawing cyano (CN) group was incorporated into the A units BT, DPQ, and DBP to afford three D–A type target polymers PB–BTCN, PB–DPQCN, and PB–DBPCN, respectively. Owing to the significant contribution of the CN-substituent, these polymers exhibit not only low-lying energy levels of both the highest occupied molecular orbital and the lowest unoccupied molecular orbital, but also reduced bandgaps. Furthermore, to investigate the photovoltaic properties of polymers, inverted-type devices with the structure of ITO/ZnO/Polymer:PC_71_BM/MoO_3_/Ag were fabricated and analyzed. All the polymer solar cells based on the three cyano-substituted conjugated polymers showed high open-circuit voltages (*V_oc_*) greater than 0.89 V, and the highest power conversion efficiency of 4.59% was obtained from the device based on PB-BtCN with a *V_oc_* of 0.93 V, short-circuit current of 7.36 mA cm^−2^, and fill factor of 67.1%.

## 1. Introduction

In the past few decades, polymer solar cells (PSCs) have attracted significant interest as a promising clean and renewable energy resource, owing to their advantages, such as high flexibility, facile processability, and cost-effectiveness [1,2,3,4]. Recent innovative efforts to improve PSCs have led to a significant enhancement, over 10%, in their power conversion efficiency (PCE) [5,6,7,8,9]. A bulk heterojunction (BHJ) configuration between polymeric electron donors and electron acceptors (usually fullerene-based materials) in an active layer of PSCs is commonly utilized, which allows the generation of bicontinuous interpenetrating networks with large interfacial areas for an efficient charge separation and charge transport process [10,11]. For further improvement of their performances, the design and synthesis of proper polymeric donor materials with strong light absorption, high charge carrier mobility, and balanced electronic structure are of great importance [12]. Usually, the alternating donor-acceptor (D–A) type copolymers between electron-donating and electron-accepting building blocks are used for PSCs, because this configuration can reduce the band gap of polymers significantly through the facile formation of the intramolecular charge transfer (ICT) state [13]. For example, the electron-donating benzodithiophene (BDT), carbazole, and triphenylamine derivatives were widely utilized for D components, whereas the electron-accepting benzothiadiazole (BT) and quinoxaline (Qx) units were often used for A moieties during the preparation of D–A type conjugated polymers for PSCs [14,15,16,17,18,19,20,21,22].

Recently, the incorporation of electron-withdrawing groups, such as fluorine atoms specifically onto the electron-accepting unit of D–A type polymers, has been regarded as a promising strategy to enhance the photovoltaic performances of PSCs [23,24,25,26,27]. Upon addition of strong electron-withdrawing groups, the energy levels of D–A type polymers, in both the lowest unoccupied molecular orbital (LUMO) and the highest occupied molecular orbital (HOMO), are lowered without a serious interruption in the bandgap. The low-lying HOMO energy level of polymeric donors can enhance the open-circuit voltage (*V_oc_*) of the device, which can also trigger a sharp increase in PCE. Along with fluorine atoms, other electron-withdrawing units, such as trifluoromethyl (–CF_3_), sulfonyl (–SO_2_), and cyano (–CN) groups, were utilized during the preparation of D–A type conjugated polymers [28,29,30]. In particular, the incorporation of the CN group into the chemical structures of D–A type polymers is beneficial for achieving high-performance PSCs, because the CN unit can not only facilitate the charge separation process in BHJ device through the generation of strong dipole moment, but also stabilize the LUMO and HOMO energy levels of polymers [30,31]. For instance, D–A type polymers with CN substituents on the electron-accepting BT and pyridine-fused triazole exhibit high PCEs of 6.55% and 8.37%, respectively [30,32].

**Figure 1:**
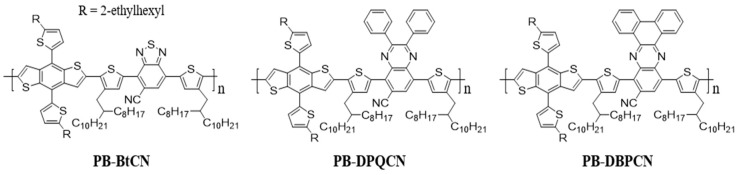
Chemical structures of PB–BtCN, PB–DPQCN, and PB–DBPCN.

Inspired by these findings, we synthesized a series of D–A type polymers, in which the electron-donating alkylthienyl-substituted BDT was connected to the electron-accepting units possessing one CN group via a thiophene bridge. Three CN-substituted strong electron-accepting moieties, i.e., BT, diphenylquinoxaline (DPQ), and dibenzophenazine (DBP), were used as building blocks for the three target polymers PB–BtCN, PB–DPQCN, and PB–DBPCN, respectively (Figure 1). Owing to the significant contributions of diverse electron-accepting moieties, discernible optical and electrochemical properties were measured from these polymers. In addition, the photovoltaic properties of the polymers were investigated by fabricating an inverted-type PSC with the structure of indium tin oxide (ITO)/ZnO/active layer (Polymer:PC_71_BM)/MoO_3_/Ag. Interestingly, the photovoltaic cells based on the three polymers show a high *V_oc_*, greater than 0.89 V, and the best PCE, of 4.59%, was obtained from the device based on PB-BtCN with a *V_oc_* of 0.93 V, a short-circuit current (*J_sc_*) of 7.36 mA cm^−2^, and a fill factor (*FF*) of 67.1%.

## 2. Experimental Section

### 2.1. Materials and Instruments

3-(2-octyldodecyl) thiophene (1), 4.7-dibromo-5-fluorobenzo[c] [1,2,5] thiadiazole (3), and 2,6-bis(trimethyltin)-4,8-bis(5-2-ethylhextyl)thiophene-2-yl)benzo[1.2-b:4,5-b’]dithiophene (9) were synthesized according to the procedure described in previous literature [33,34,35]. (6,6)-Phenyl C_71_ butyric acid methyl ester (PC_71_BM) and (6,6)-Phenyl C_61_ butyric acid methyl ester (PC_61_BM) were purchased from Nano-C, Inc.(Westwood, USA) All other chemicals and solvents were obtained from Aldrich. ^1^H and ^13^C nuclear magnetic resonance (NMR) spectra were analyzed using a JEOL JNM ECP-600 spectrometer (Tokyo, Japan). UV–visible spectra were measured using a JASCO V-530 UV-Vis spectrophotometer (Eastony, USA). Matrix-assisted laser desorption/ionization time-of-flight spectroscopy was performed using a Bruker Ultraflex spectrometer (Billerica, USA). Gel permeation chromatography (GPC) was performed using an Agilent 1200 series instrument (Santa Clara, USA). Cyclic voltammetry (CV) measurements were obtained using a VersaSTAT3 potentiostat (Princeton Applied Research, Oak Ridge, USA) with tetrabutylammonium hexafluorophosphate (0.1M in acetonitrile) as the electrolyte. A glassy carbon electrode coated with polymers, a platinum wire, and a silver wire were used as the working electrode, counter electrode, and pseudo-reference electrode with a ferrocene/ferrocenium external standard, respectively. The inverted-type PSCs, with a configuration of ITO/ZnO/active layer (Polymer:PC_71_BM)/MoO_3_/Ag, were sequentially fabricated and analyzed by following a previously reported procedure [36]. In particular, to fabricate inverted type photovoltaic devices with indium tin oxide (ITO)/Zinc oxide (ZnO)/active layer (donor:acceptor)/MoO_3_/Ag, a 25 nm-thick ZnO film was deposited on an ITO surface by using a sol–gel process. The partially crystalline ZnO film was prepared by thermal curing of pre-deposited ZnO precursors at 200 °C for 10 min. The solution of ZnO precursors was prepared by the dissolution of zinc acetate dehydrate (0.1 g) and ethanolamine (0.025 mL) in methoxyethanol (1 mL) and stirring the mixture at 60 °C for 12 h prior to film deposition. Prior to spin-coating, the solution of ZnO precursors was filtered through a 0.45 μm cellulose acetate membrane filter. The active layer was spin-coated by the chlorobenzene (with 1, 8-diiodooctane) solution of polymeric donor and PCBM acceptor. The solution was prepared by a concentration of 10 mg/mL based on a polymeric donor and blended at 70 °C for 12 h. Prior to spin-coating, the blended solution was filtered through a 0.2 μm polytetrafluoroethylene membrane filter. Finally, a 3 nm thick MoO_3_ layer and 100 nm thick Ag layer were consecutively deposited by thermal evaporation at 1.5 × 10^−6^ Torr through a shadow mask with a device area of 0.12 cm^2^. The *J*–*V* characteristics of devices were analyzed by using a KEITHLEY Model 2400 source-measure unit, under AM 1.5G illumination at 100 mW/cm^2^ from a 150 W Xe lamp. The conditions of a solar simulation were calibrated before the measurements by using a Si reference cell with a KG5 filter certified by the National Institute of Advanced Industrial Science and Technology. Atomic force microscopy (AFM) images were obtained using a Bruker (NanoScope V, Billerica, USA) micropore in tapping mode.

### 2.2. Syntheses of Monomers and Polymers

#### 2.2.1. Synthesis of (4-(2-Octyldodecyl)thiophen-2-yl)tributylstannane (2)

After dissolving 3-(2-octyldodecyl)thiophene (1, 8.23 mmol) in tetrahydrofuran (THF, 20 mL), n-butyllithium (8.23 mmol, 2.5 M in hexane) was slowly added at −78 °C under a N_2_ atmosphere. The solution was stirred for 1 h and, subsequently, tributylchlorostannane (8.23 mmol) was added. The mixture was stirred for 1 h at −78 °C and, thereafter, further reacted overnight at room temperature. Once the reaction was completed, the mixture was poured into water and extracted with hexane. The organic layer was dried over magnesium sulfate (MgSO_4_). The solvents were removed under reduced pressure and, thereafter, the product (2) was used directly for the subsequent step without further purification.

#### 2.2.2. Synthesis of 5-Fluoro-4,7-bis(4-(2-octyldodecyl)thiophen-2-yl)benzo[c][1,2,5]thiadiazole (4)

4,7-dibromo-5-fluorobenzo[c][1,2,5]thiadiazole (3, 2.50 mmol), 4-(2-octyldodecyl)thiophen-2-yl)tripropylstannane (2, 5.50 mmol), and Pd(P(Ph_2_)Cl_2_)(5 mol %) were dissolved in toluene (30 mL). After N_2_ had been bubbled for 30 min, the mixture was heated to reflux and stirred for 24 h under a N_2_ atmosphere. After the reaction was completed, the mixture was cooled to room temperature and filtered with celite to remove the palladium catalyst. The residue was purified via column chromatography using n-hexane. Yield:46%. ^1^H NMR (600 MHz, CDCl_3_): δ (ppm) = 8.07 (s, 1H), 7.95 (d, 1H), 7.74 (d, 1H), 7.12 (s, 1H), 7.06 (s, 1H), 2.64 (dd, 4H), 1.69(d, 2H), 1.32–1.24 (m, 64H), 0.87 (t, 12H). ^13^C NMR (600 MHz, CDCl_3_): δ (ppm) = 159.68, 158.00, 153.44, 149.74, 143.16, 142.06, 137.49, 132.09, 130.37, 125.76, 123.88, 123.59, 116.49, 111.23, 39.04, 35.07, 33.47, 32.10, 30.23, 29.87, 29.56, 26.80, 14.14.

#### 2.2.3. Synthesis of 4,7-Bis(5-bromo-4-(2-octyldodecyl)thiophen-2-yl)-5- fluorobenzo[c][1,2,5]thiadiazole (5)

A mixture of 5-fluoro-4,7-bis(4-(2-octyldodecyl)thiophen-2-yl)benzo[c][1,2,5]thiadiazole (4; 0.91mmol) and *N*-bromosuccinimide (NBS, 2.73 mmol) in THF (30 mL) was stirred at room temperature for 12 h. The solution was poured into water and extracted with ethyl acetate. The organic layer was dried with MgSO_4_ and filtered. The solvents were removed under reduced pressure and the residue was purified via column chromatography using n-hexane. Yield:50%. ^1^H NMR (600 MHz, CDCl_3_): δ (ppm) = 7.93 (s, 1H), 7.73 (s, 1H), 7.66 (d, 1H), 2.58 (dd, 4H), 1.75 (d, 2H), 1.35–1.23 (m, 64H), 0.86 (t, 12H). ^13^C NMR (600 MHz, CDCl_3_): δ (ppm) = 159.44, 157.76, 152.66, 149.10, 142.27, 141.32, 136.71, 131.92, 131.45, 129.15, 124.59, 115.50, 113.85, 110.42, 38.53, 34.04, 33.34, 31.95, 30.07, 29.74, 29.40, 26.55, 22.71,14.27.

#### 2.2.4. Synthesis of 4,7-Bis(5-bromo-4-(2-octyldodecyl)thiophen-2-yl)benzo[c][1,2,5]thiadiazole-5-carbonitrile (6)

A mixture of 4,7-bis(5-bromo-4-(2-octyldodecyl)thiophen-2-yl)-5-fluorobenzo[c][1,2,5]thiad iazole (4, 0.60 mmol), KCN (1.98 mmol) and 18-crown-6 (0.20 mmol) was dissolved in a mixed solvent of toluene (20 mL) and dimethylformamide (DMF, 4 mL). After N_2_ had been bubbled for 30 min, the mixture was stirred and refluxed for 48 h. After the reaction was completed, the mixture was poured into water and extracted with methylene chloride. Ammonia solution (28%) was poured into the aqueous extracts to remove the residual cyanide. The organic phase was dried over MgSO_4_. The product was purified via column chromatography using n-hexane/methylene chloride (20/1) (*v*/*v*). Yield:60%. ^1^H NMR (600 MHz, CDCl_3_): δ (ppm) = 7.93 (s, 1H), 7.89 (s, 1H), 7.74 (s, 1H), 2.59 (dd, 4H), 1.75 (s, 2H), 1.31–1.23 (m, 64H), 0.89–0.84 (m, 12H). ^13^C NMR (600 MHz, CDCl_3_): δ (ppm) = 152.55, 152.45, 142.63, 142.07, 136.18, 133.79, 132.34, 129.62, 129.51, 126.75, 125.86, 118.68, 117.32, 114.26, 108.15, 38.54, 34.22, 34.12, 33.31, 31.90, 29.99, 29.65, 29.62, 29.34, 26.52, 22.67,14.11. MALDI-TOF MS:m/z calculated, 1044.292; found, 1043.544 [M^+^].

#### 2.2.5. Synthesis of 5,8-Bis(5-bromo-4-(2-octyldodecyl)thiophen-2-yl)-2,3-diphenylquinoxaline-6- carbonitrile (7)

A mixture of 7-bis(5-bromo-4-(2-octyldodecyl)thiophen-2-yl)benzo[c][1,2,5]thiadiazole-5-carbonitrile (6, 0.32 mmol) and zinc powder (6.40 mmol) was stirred in acetic acid (15 mL) at reflux temperature for 3 h. After the reaction was completed, zinc powder was filtered and the filtrate was collected. Benzil (0.32 mmol) was added to the filtrate and stirred overnight at reflux. After cooling to room temperature, the mixture was poured into water and extracted with ethyl acetate. The organic layers were collected and dried over MgSO_4_. The solvents were removed under reduced pressure. The crude residue was further purified via column chromatography n-hexane/methylene chloride (20/1) (*v*/*v*). Yield:43%. ^1^H NMR (600 MHz, CDCl_3_): δ (ppm) = 8.26 (s, 1H), 7.83 (s, 1H), 7.73 (d, 2H), 7.68 (d, 2H), 7.52(s, 1H), 7.46–7.37 (m, 6H), 2.58 (dd, 4H), 1.76 (s, 2H), 1.33–1.23 (m, 64H), 0.86–0.84 (m, 12H). ^13^C NMR (600 MHz, CDCl_3_): δ (ppm) = 153.74, 153.24, 140.87, 137.92, 137.43, 137.40, 137.18, 135.31, 134.43, 133.17, 131.80, 131.32, 130.48, 130.41, 129.89, 129.73, 128.50, 128.45, 128.40, 127.95, 119.30, 117.98, 116.62, 109.53, 38.67, 38.61, 34.23, 34.16, 33.35, 31.91. 30.01, 29.68, 29.66, 29.34, 26.57, 22.68, 14.12. MALDI-TOF MS: m/z calculated, 1190.450; found, 1190.682 [M^+^]

#### 2.2.6. Synthesis of 10,13-Bis(5-bromo-4-(2-octyldodecyl)thiophen-2-yl)dibenzo[a,c]phenazine-11-carbonitrile (8)

The synthetic procedure of the product 8 was the same as the method for the preparation of the product 7 described above. 9,10-Phenantherenequinone was used instead of benzil with the same equivalence. Yield:48%. ^1^H NMR (600 MHz, CDCl_3_): δ (ppm) = 9.51 (d, 1H), 9.39 (d, 1H), 8.60 (t, 2H), 8.32 (s, 1H), 7.90–7.82 (m, 5H), 7.57 (s, 1H), 2.65 (dd, 4H) 1.83 (s, 2H) 1.38–1.23 (m, 64H), 0.86–0.82 (m, 12H). ^13^C NMR (600 MHz, CDCl_3_): δ (ppm) = 141.88, 141.65, 140.45, 140.22, 137.50, 136.87, 134.55, 133.31, 133.06, 131.90, 131.65, 131.40, 130.88, 130.58, 129.81, 128.57, 128.35, 127.78, 127.65, 127.06, 126.67, 122.19, 119.47, 118.47, 116.79, 108.14, 38.87, 34.32, 34.26, 33.52, 33.49, 32.12, 32.09, 32.06, 30.33, 29.91, 29.87, 29.84, 29.55, 29.52, 26.79, 26.74, 22.86, 22.83, 22.81, 14.25. MALDI-TOF MS:m/z calculated, 1088.433; found, 1088.701 [M^+^].

#### 2.2.7. Synthesis of PB-BtCN Via Palladium-Catalyzed Stille Reaction

A mixture of 4,7-bis(5-bromo-4-(2-octyldodecyl)thiophen-2-yl)benzo[c][1,2,5]thiadiazole-5-carbonitrile (6, 0.12 mmol), 2,6-bis(trimethyltin)-4,8-bis(5-2-ethylhextyl)thiophene-2-yl) benzo[1,2-b:4,5-b′]dithiophene (9, 0.12 mmol), and Pd(PPh_3_)_4_ (3 mol %) was dissolved in dry toluene (5 mL). After N_2_ has been bubbled for 30 min, the mixture was stirred at 90 °C under N_2_ atmosphere for 48 h. At the end of polymerization, 2-tributylstannylthiophene and 2-bromothiophene were subsequently added, with an interval of 2 h. Once the polymerization was completed, the mixture was cooled to room temperature and precipitated into methanol. The black solids were gathered and further purified via Soxhlet extraction with methanol, acetone, hexane, and chloroform, in sequence. The final solution was collected and the solvent was evaporated to concentrate the polymer solution. Subsequently, the polymers were precipitated again using methanol. Finally, the polymer was dried overnight in vacuum oven at 50 °C. Yield: 88%. ^1^H NMR (600 MHz, CDCl_3_): δ (ppm) = 8.08–7.97 (br, 2H), 7.82–7.80 (br, 2H), 7.48–7.43 (br, 1H), 7.38–7.34 (br, 2H), 7.10–7.08 (br, 1H), 6.98–6.94 (br, 1H), 3.02–2.90 (br, 8H), 1.80–1.73 (br, 4H), 1.45–1.43 (br, 16H), 1.31–1.19 (br, 64H), 0.98–0.92 (br, 12H), 0.85–0.83 (br, 12H). Molecular weight by GPC: number-average molecular weight (M_n_) = 18.52 KDa, polydispersity index (PDI) = 2.20. Elemental analysis: calculated (%) for C: 73.04, H: 8.75, N: 2.87, found: C: 72.71, H: 8.46, N: 2.65.

#### 2.2.8. Synthesis of PB–DPQCN

The synthetic procedure of PB-DPQCN was the same as the method for the preparation of PB–BtCN described above. Compounds 7 and 9 were used as monomers. Yield: 84%. ^1^H NMR (600 MHz, CDCl_3_): δ (ppm) = 8.34 (br, 1H), 8.07 (br, 2H), 7.88–7.85 (br, 8H), 7.44–7.43 (br, 3H), 7.40–7.38 (br, 4H), 7.00 (br, 2H), 2.93 (br, 8H), 1.86–1.85 (br, 2H), 1.77–1.75 (br, 2H), 1.35 (br, 16H), 1.28–1.21 (br, 64H), 0.92–0.89 (br, 12H), 0.84 (br, 12H). Yield: 70%. Molecular weight by GPC: M_n_ = 25.47 KDa, PDI = 1.54. Elemental analysis: calculated (%) for C: 76.86, H: 8.58, N: 2.61, found: C: 76.86, H: 8.36, N: 2.45.

#### 2.2.9. Synthesis of PB–DBPCN

The synthetic procedure of PB–DBPCN was the same as the method for the preparation of PB–BtCN, described above. Compounds 8 and 9 were used as monomers. Yield: 89%. ^1^H NMR (600 MHz, CDCl_3_): δ (ppm) = 8.64–8.52 (br, 1H), 8.34–8.22 (br, 1H), 8.17–8.1 (br, 2H), 7.92–7.82 (br, 4H), 7.69–7.55 (br, 4H), 7.43–7.36 (br, 1H), 7.23–7.15 (br, 1H), 7.08–7.03 (br, 3H), 3.07–2.79 (br, 8H), 1.88–1.84 (br, 4H), 1.4–1.35 (br, 16H), 1.28–1.22 (br, 64H), 0.98–0.89 (br, 12H), 0.84–0.80 (br, 12H). Yield: 95%. Molecular weight by GPC: M_n_ = 12.48 KDa, PDI = 2.41. Elemental analysis: calculated (%) for C: 76.95, H: 8.46, N: 2.61, found: C: 77.04, H: 8.28, N: 2.52.

## 3. Results and Discussion

### 3.1. Synthesis and Thermal Properties

**Scheme 1:**
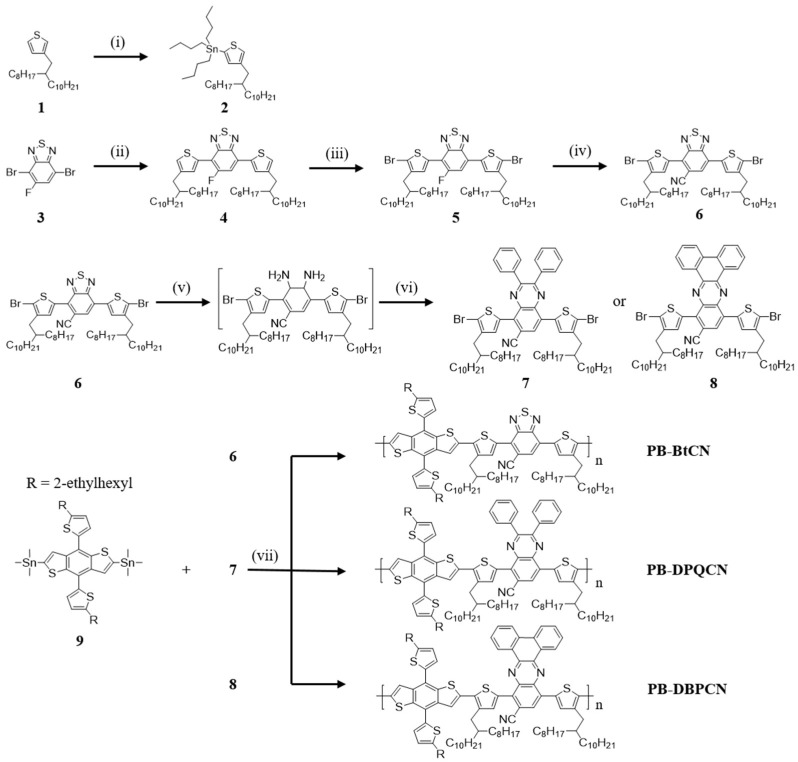
Synthetic scheme of the monomers and polymers. (**i**) N-butylithium, THF, room temperature, overnight; N_2_; (**ii**) Pd(P(Ph_2_)Cl_2_), toluene, reflux, 48 h, N_2_; (**iii**) NBS, room temperature, overnight; (**iv**) 18-crown-6, KCN, THF, DMF, reflux, 48 h, N_2_; (**v**) zinc, acetic acid, 80 °C, 3 h (**vi**) benzil or 9,10-phenantherenequinone , acetic acid, reflux; and(**vii**) Pd(PPh_3_)_4_, toluene, 90 °C, 48 h.

**Figure 2:**
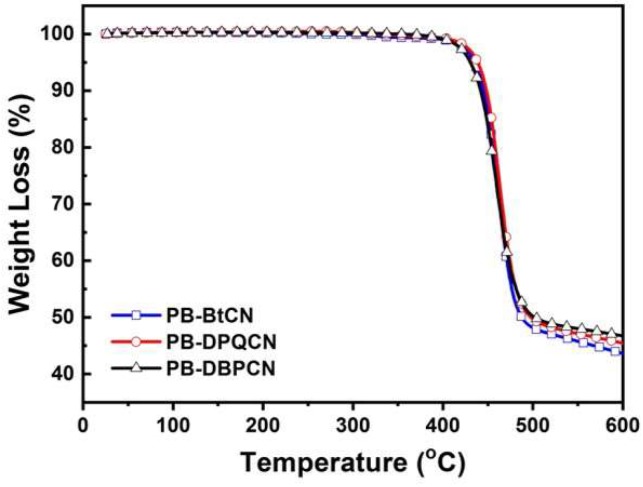
TGA curves of the polymers under N_2_ at a heating rate of 10 °C/min.

The overall synthetic routes of the monomers and polymers are shown in Scheme 1. First, compound 1 and tributylchlorostannane were reacted in the presence of n-butyllithium to produce compound 2. Second, Stille reaction between compounds 2 and 3 yielded compound 4 and the following bromination reaction of compound 4, using NBS, readily afforded the dibrominated BT compound of 5. Third, the CN-substituted BT-based monomer 6 was obtained by replacing fluorine atoms in compound 5 with the CN group. In addition to the BT-based monomer, two reactions, i.e., a zinc (Zn)-mediated reduction of compound 6 and a condensation reaction of *ortho*-diamine intermediated with the related *α*-diketone, were consecutively conducted to produce DPQ- and DBP-based monomers. The condensation reaction of benzil and 9,10-phenantherenequinone produced two additional cyano-containing monomers, 7 and 8, respectively. Finally, polymerization reactions between the alkylthienyl-substituted BDT-based electron-donating monomer 9 and the electron-accepting CN-substituted monomers 6, 7, and 9 under Stille polycondensation condition yielded three D–A type target polymers, PB–BtCN, PB–DPQCN, and PB–DBPCN, respectively. Owing to the existence of multiple alkyl chains, these polymers exhibit good solubility in organic solvents, such as chloroform, THF, and toluene. GPC measurement in THF displays the number average molecular weight (M_n_) values of 18.52, 25.47, and 12.48 KDa for PB–BtCN, PB–DPQCN, and PB–DBPCN, respectively, with the corresponding polydispersity indexes of 2.20, 1.54, and 2.41, respectively. In addition, thermogravimetric analysis (TGA) of all the polymers at a heating rate of 10 °C/min, under N_2_, shows good thermal stability with a similar high onset of decomposition temperature, at 5% weight loss (*T*_d5%_), of approximately 420 °C (Figure 2).

### 3.2. Optical and Electrochemical Properties

**Figure 3:**
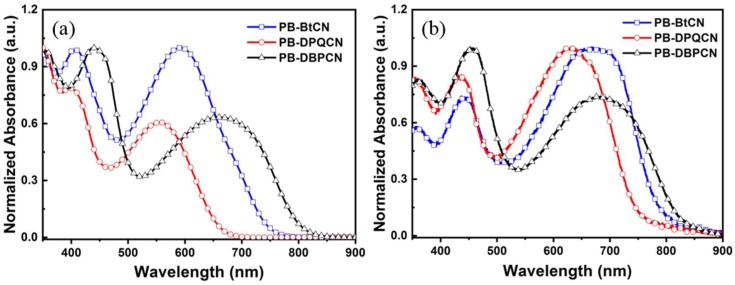
UV–Vis spectra of PB-BtCN, PB-DPQCN, and PB-DBPCN (**a**) in chloroform solution and (**b**) in thin films (as for film sample, spectra are offset for clarity).

Similar to other D–A type polymers [36,37,38], the three CN-substituted polymers exhibit typical two broad absorption bands (Figure 3). The absorption bands at the shorter wavelength are related to the π–π* electron transition of the conjugated backbones, whereas those at the longer wavelength are related to the ICT state between the D and A units of the polymers. The absorption maximums of PB–BtCN, PB–DPQCN, and PB–DBPCN at the ICT region in a chloroform solution were observed at 594, 557, and 662 nm, respectively (Figure 3a). The maximum ICT absorption peaks of all the polymers in the film were red-shifted to 670, 629, and 682 nm, respectively, compared with those in the solution (Figure 3b), which is attributed to the aggregation and ordering assembly of polymer chains in the solid state. In addition, the absorption spectra of PB–BtCN, PB–DPQCN, and PB–DBPCN show discernible patterns with different maximum values in both shorter- and longer-wavelength regions, depending on the A units of the polymer backbone. The change from BT to DBP with more planar phenanthrene moiety induces red-shifted absorption spectra, owing to the extension of the effect conjugated length. However, the transition from BT to DPQ displays a blue shift of the absorption spectra, owing to the reduction of conjugation length caused by the existence of the twisted two benzene rings at 2,3-positions of the quinoxaline unit [39].

**Figure 4:**
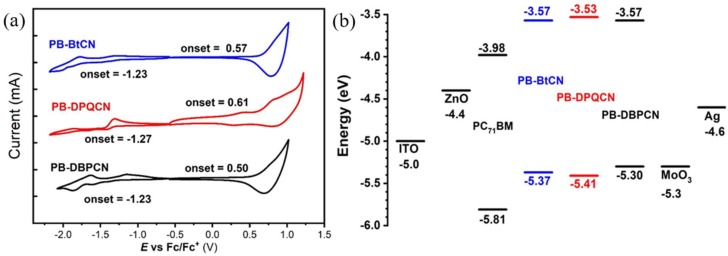
(**a**) CV measurements of the polymers and (**b**) the energy level diagram of the polymers and materials used in this study.

The HOMO energy levels of PB–BtCN, PB–DPQCN, and PB–DBPCN were determined by the onset of oxidation potential of the CV measurement following the relationship of *E_HOMO_* = −(*E_ox_* + 4.8 eV) and the values were −5.37, −5.41, and −5.30 eV, respectively (Figure 4a). The LUMO energy levels of PB–BtCN, PB–DPQCN, and PB–DBPCN were also calculated from the onset of reduction potential of the CV curve, using the equation *E_LUMO_* = −(*E_re_* + 4.8 eV), and the values were −3.57, −3.53, and −3.57 eV, respectively. Therefore, the bandgaps of PB–BtCN, PB–DPQCN, and PB–DBPCN were determined from the HOMO and LUMO energy levels to be 1.80, 1.88, and 1.73 eV, respectively. All the polymers exhibit relatively low-lying HOMO and LUMO energy levels, owing to the presence of CN-substituents in their structures. The optical and electrochemical characteristics of PB–BtCN, PB–DPQCN, and PB–DBPCN are summarized in Table 1. In addition, the energy level diagrams of the polymers, PC_71_BM, and other components required for fabricating an inverted-type PSC are demonstrated in Figure 4b. Based on this energy level diagram, the facile operation of photovoltaic devices is expected through the energetically favored charge separation and transport process.

### 3.3. Photovoltaic Properties

**Figure 5:**
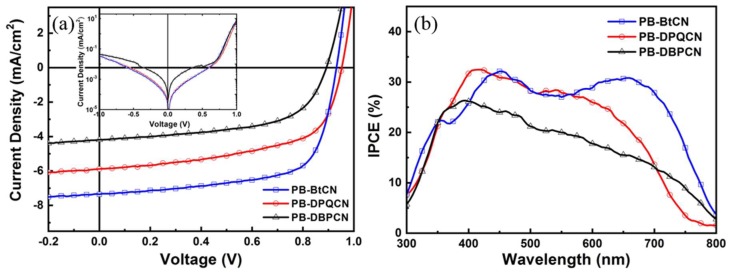
(**a**) Current density–voltage (*J*–*V*) curves of the PSCs and (**b**) the IPCE spectra of the PSCs based on PB–BtCN, PB–DPQCN, and PB–DBPCN.

To determine the photovoltaic properties of the polymers, inverted-type PSCs, with a configuration of ITO/ZnO (25 nm)/Polymer:PC_71_BM(80 nm)/MoO_3_ (20 nm)/Ag (100 nm), were fabricated. Various blend ratios between the polymer and PC_71_BM in the active layer were tested with 3 wt % 1.8-diiodooctane as a processing additive and the optimum blend ratios of 3:6, 3:5, and 3:4 were determined for the devices with PB–BtCN, PB–DBPCN, and PB–DPQCN, respectively (Tables S1 and S2 in Electronic Supporting Information (ESI)). In this study, PC_71_BM was used as an acceptor due to its better performances in BHJ photovoltaic cells, compared to the devices with PC_61_BM (Table S2, ESI) [40,41]. Figure 5a shows the current density–voltage (*J*–*V*) curves of the PSCs at the optimized blend ratio, under AM 1.5G condition. Owing to the strong electron-withdrawing CN substituent in the polymer backbone, all the PSCs based on three polymers show a high *V_oc_* greater than 0.89 V. The *V_oc_* values of the devices with PB–BtCN, PB–DPQCN, and PB–DBPCN are 0.93, 0.95, and 0.89 V, respectively, which is consistent with the trend in their HOMO energy levels (vide supra). Moreover, the *J_sc_* values of the PSCs are gradually decreased in the order of PB–BtCN, PB–DPQCN, and PB–DBPCN, with the values of 7.36, 5.89, and 4.19 mA/cm^2^, respectively. The CN-substituted D–A type polymer with BT as an electron-accepting unit could induce a significant increase in the *J_sc_* value of the PSCs, approximately 25% and 75% higher than those with DPQ and DBP moieties, respectively, at the same position. Similarly, the device based on PB–BtCN exhibited the highest *FF* value of 67.1%, whereas the corresponding values for the devices with PB–DPQCN and PB–DBPCN were limited to 58.8% and 58.3%, respectively. It is observed, from the *V_oc_*, *J_sc_*, and *FF* values obtained from the *J*–*V* curves, that the best PCE, of 4.59%, was achieved for the device based on PB–BtCN, rather than for those based on DPQCN (3.29%) and PB–DBPCN (2.17%). As shown in Figure 5b, the incident photon-to-current efficiency (IPCE) curves of all the devices exhibited good response within the wavelength range 300–800 nm. As expected, the IPCE of the device based on PB–BtCN was higher than those of the other devices fabricated with PB–DPQCN and PB–DBPCN, particularly in the longer wavelength region (>600 nm). Therefore, this result confirms the highest *J_sc_* value for the device based on PB–BtCN. In addition, all the *J_sc_* values calculated from the IPCE curves are consistent with those observed from the *J*–*V* curves. Furthermore, the series resistance (*R_s_*) of the devices was obtained from the *J*–*V* curves under dark condition (inset of Figure 5a). The *R_s_* of the device based on PB–BtCN was 3.75 Ω cm^2^, which was smaller than those of the devices based on PB–DPQCN (4.34 Ω cm^2^) and PB–DBPCN (9.96 Ω cm^2^). It is evident that the *R_s_* values of the devices are also strongly correlated with the trend in the photovoltaic properties of the polymers in inverted-type PSCs. The photovoltaic parameters are summarized in Table 2.

**Figure 6:**
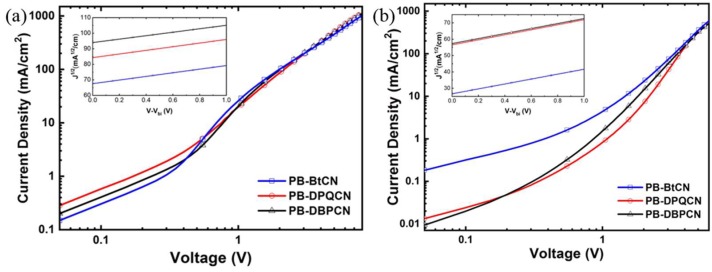
Current density vs. voltage (**a**) hole- and (**b**) electron- devices (inset: current density vs. voltage—built-in voltage (V_bi_) curves with fitted lines) based on PB–BtCN, PB–DPQCN, and PB–DBPCN.

Furthermore, the charge transporting properties of the polymers were analyzed by fabricating hole- and electron-only devices with the structures of ITO/PEDOT: PSS/Polymer:PC_71_BM/Au and ITO/ZnO/Polymer:PC_71_BM/LiF/Al, respectively. As shown in Figure 6, the *J*–*V* curves of all the polymers show a typical space charge limited current behavior governed by the Mott–Gurney law [42]. The calculated hole mobilities of the devices based on PB–BtCN, PB–DPQCN, and PB–DBPCN are 4.66 × 10^−3^, 2.16 × 10^−3^, and 1.96 × 10^−3^ cm^2^ V^−1^ s^−1^, respectively, whereas their electron mobilities are 4.98 × 10^−3^, 2.07 × 10^−3^, and 2.09 × 10^−3^, respectively. Interestingly, the devices based on PB–BtCN exhibit more than two times higher values of both hole- and electron-mobility than those based on PB–DPQCN and PB–DBPCN. These results can confirm the higher *J_sc_* and *FF* values of the PSC based on PB–BtCN, compared with those of the devices based on PB–DPQCN and PB–DBPCN.

**Figure 7:**
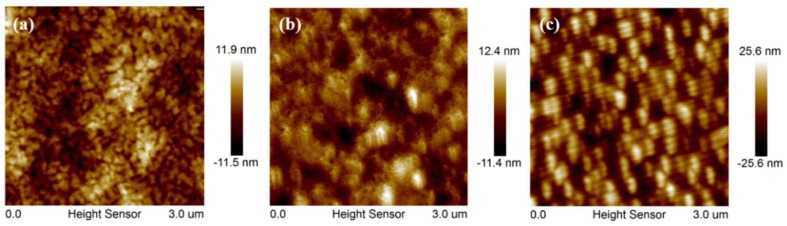
AFM images of the active layers based on (**a**) PB–BtCN, (**b**) PB–DPQCN, and (**c**) PB–DBPCN.

The surface morphologies of the active layers in the PSCs are of great importance to understand the photovoltaic performances of the devices. AFM measurement in tapping mode was conducted to determine the surface characteristics of the blended films, composed of polymers with PC_71_BM, and the results are shown in Figure 7. The root-mean-square (RMS) values of the films based on PB–BtCN, PB–DPQCN, and PB–DBPCN are 2.72, 2.50, and 6.88 nm, respectively. The high RMS value of the active layer based on PB–DBPCN is ascribed to large aggregation, owing to its poor miscibility with PC_71_BM (Figure 7c). However, those based on PB–BtCN and PB–DPQCN exhibit relatively uniform and smoother morphology without noticeable aggregations, which can lead to the enhancement of the *J_sc_* and *FF* of PSCs through efficient charge dissociation and transport, together with a good interfacial contact [43].

## 4. Conclusions

In this study, a series of conjugated polymers, in which the electron-donating BDT moiety was connected to the CN-substituted electron-accepting group via a thiophene bridge, were synthesized under the Stille coupling condition. To clarify their effects on diverse properties of the polymers, three distinct units, BT, DPQ, and DBP, were used as an electron-accepting component to afford three target polymers, PB–BtCN, PB–DPQCN, and PB–DBPCN, respectively. Owing to the significant contributions of the CN moiety on the electron-accepting units of the polymers, all the polymers exhibit low-lying energy levels of HOMO and LUMO and reduced bandgaps. The photovoltaic properties of the polymers were investigated by constructing inverted-type devices with the structure of ITO/ZnO/Polymer:PC_71_BM/MoO_3_/Ag. The low-lying HOMO energy levels of the polymers led to a high *V_oc_* greater than 0.89 V and the highest PCE, of 4.59%, was achieved for the device based on PB-BtCN, rather than for the devices with PB–DPQCN (3.29%) and PB–DBPCN (2.17%). The better incident photon-to-current response at the longer-wavelength region, and higher hole and electron mobilities with a low *R_s_* value for the device based on PB–BtCN, can efficiently improve the *J_sc_* and *FF* values of the PSC. These findings suggest that the type of A unit in the CN-substituted D–A type polymers plays a crucial role for determining the photovoltaic properties of the PSCs. Therefore, this study can pave the way for optimizing the structure-property relationships of CN-substituted D–A type conjugated polymers for photovoltaic cells.

## Figures and Tables

**Table 1:** Summary of the optical and electrochemical properties of the polymers.

Polymer	$\lambda_{max}^{solution}\left(nm\right){\;}^{a}$ $,\;(\lambda_{max}^{film}\left(nm\right){\;}^{b})$	$\mathrm{HOMO}\;\left(eV\right){\;}^{c}$	$\mathrm{LUMO}\;\left(eV\right){\;}^{d}$	$\mathrm{Bandgap}\;\left(eV\right){\;}^{e}$
PB-BtCN	406, 594, (442, 670)	−5.37	−3.57	1.80
PB-DPQCN	391, 557, (434, 629)	−5.41	−3.53	1.88
PB-DBPCN	441, 662, (452, 682)	−5.30	−3.57	1.73

^a^ Maximum wavelength of the solution; ^b^ Maximum wavelength of the film; ^c^ Estimated from the oxidation onset potential; ^d^ Estimated from the reduction onset potential; ^e^ Determined by subtracting the highest occupied molecular orbital (HOMO) energy from that of the lowest unoccupied molecular orbital (LUMO).

**Table 2:** Best photovoltaic parameters of the devices based on the polymer. The averages of the photovoltaic parameters of each device are shown in parentheses.

Polymer	Blend Ratio	*J*_sc_ (mA/cm^2^)	$V_{\mathrm{oc}}\;(V)$	FF (%)	PCE (%)	*R_s_^a^* (Ω cm^2^)
PB–BtCN	3:6	7.36 (7.24 ± 0.18)	0.93 (0.94 ± 0.00)	67.1 (65.1 ± 1.51)	4.59 (4.40 ± 0.10)	3.75
PB–DPQCN	3:5	5.89 (5.89 ± 0.09)	0.95 (0.94 ± 0.01)	58.8 (57.2 ± 0.95)	3.29 (3.17 ± 0.07)	4.34
PB–DBPCN	3:4	4.19 (4.09 ± 0.13)	0.89 (0.89 ± 0.01)	58.3 (56.9 ± 0.72)	2.17 (2.07 ± 0.08)	9.96

^a^ Series resistance (estimated from the corresponding best device).

## Supplementary Materials

The following are available online at https://www.mdpi.com/2073-4360/11/5/746/s1, Table S1: the best photovoltaic parameters of the PSCs, Table S2: the photovoltaic parameters of the devices based on PB-BtCN.
