# Supplementary material for: Synthesis of Cyano-Substituted Conjugated Polymers for Photovoltaic Applications
Source: Polymers (Basel). 2019 Apr 26;11(5):746. doi: 10.3390/polym11050746 (PMC6571826; doi:10.3390/polym11050746)
Supplement: Supplementary file 1 [file polymers-11-00746-s001.pdf]

Electronic Supporting Information (ESI) for:

Synthesis of Cyano-Substituted Conjugated Polymers for Photovoltaic Applications

Mun Ho Yang [1,†], Ho Chel Jin[2,†], Joo Hyun Kim[2,*] and Dong Wook Chang [1,*]

[1] Department of Industrial Chemistry, and [2]Department of Polymer Engineering, Pukyong National University, 48547 Busan, Republic of Korea.

Corresponding: jkim@pknu.ac.kr; dwchang@pknu.ac.kr

[†] These authors contributed equally.

Table S1. The best photovoltaic parameters of the PSCs. The average and deviation (10 devices are averaged) for the photovoltaic parameters of each device are given in parentheses.

Donor	Blend ratio[a]	J_{sc} (mA/cm^2)	V_{oc} (V)	*FF* (%)	*PCE* (%)	R_s (Ω cm^2)[b]
**PB-BtCN**	3:3	6.26 (6.01)	0.94 (0.94)	66.8 (65.2)	3.93 (3.69)	
	3:4	6.92 (6.65)	0.94 (0.94)	62.1 (61.8)	4.04 (3.84)	
	3:5	6.39 (6.24)	0.94 (0.94)	67.8 (68.0)	4.07 (3.99)	
	**3:6**	**7.36 (7.24)**	**0.93 (0.94)**	**67.1 (65.1)**	**4.59 (4.40)**	**3.75**
	3:7	6.99 (6.87)	0.93 (0.93)	64.0 (63.4)	4.16 (4.06)	
**PB-DPQCN**	3:2	2.76 (2.59)	0.76 (0.72)	50.9 (49.0±	1.07 (0.92)	
	3:3	5.02 (4.71)	0.90 (0.88)	58.2 (56.9)	2.63 (2.36)	
	3:4	5.75 (5.63)	0.95 (0.94)	58.6 (57.4)	3.20 (3.08)	
	**3:5**	**5.89 (5.83)**	**0.95 (0.95)**	**58.8 (57.4)**	**3.29 (3.16)**	**4.34**
	3:6	5.83 (5.79)	0.96 (0.96)	57.2 (54.3)	3.20 (3.01)	
**PB-DBPCN**	3:3	3.71 (3.63)	0.89 (0.89)	55.4 (56.0)	1.83 (1.81)	
	**3:4**	**4.19 (3.84)**	**0.89 (0.89)**	**58.3 (56.1)**	**2.17 (1.91)**	**9.96**
	3:5	3.08 (2.95)	0.89 (0.89)	51.3 (49.1)	1.41 (1.30)	
	3:6	2.71 (2.64)	0.90 (0.89)	50.0 (49.6)	1.22 (1.17)	

[a]Mass ratio of polymer donor to $PC_{71}BM$. [b]Series resistance (estimated from the corresponding best device).

Table S2. The photovoltaic parameters of the devices based on PB-BtCN with diverse concentrations of DIO additive and different type of acceptors. The averages of the photovoltaic parameters of each device are shown in parentheses.

Polymer	Blend ratio	*DIO amount*	J_{sc} (mA/cm^2)	V_{oc} (V)	*FF* (%)	*PCE* (%)
PB-BtCN : $PC_{71}BM$	3:6	2%	8.73 (8.51±0.21)	0.90 (0.89±0.01)	53.7 (51.6±0.82)	4.22 (3.91±0.25)
PB-BtCN : $PC_{71}BM$	3:6	3%	7.36 (7.24±0.11)	0.93 (0.94±0.01)	67.1 (65.1±0.95)	4.59 (4.40±0.15)
PB-BtCN : $PC_{71}BM$	3:6	4%	6.97 (6.96±0.09)	0.90 (0.90±0.01)	59.0 (56.9±1.24)	3.70 (3.56±0.17)
PB-BtCN : $PC_{61}BM$	3:6	3%	4.28 (4.01±0.26)	0.88 (0.88±0.01)	55.7 (54.3±1.49)	2.10 (2.03±0.12)
